# Human umbilical cord mesenchymal stem cells reduce systemic inflammation and attenuate LPS-induced acute lung injury in rats

**DOI:** 10.1186/1476-9255-9-33

**Published:** 2012-09-13

**Authors:** Jianjun Li, Dong Li, Xiaomei Liu, Shuhai Tang, Fengcai Wei

**Affiliations:** 1 Department of Anesthesiology, Shandong University, Shandong, PR China; 2 Cryomedicine Laboratory, Shandong University, Shandong, PR China; 3 Department of Stomatology, Qilu Hospital, Shandong University, Ji’nan, Shandong, 250012, PR China

**Keywords:** Acute lung injury, Umbilical cord, Mesenchymal stem cells, Inflammation, Heme oxygenase-1

## Abstract

**Background:**

Mesenchymal stem cells (MSCs) possess potent immunomodulatory properties and simultaneously lack the ability to illicit immune responses. Hence, MSCs have emerged as a promising candidate for cellular therapeutics for inflammatory diseases. Within the context of this study, we investigated whether human umbilical cord-derived mesenchymal stem cells (UC-MSCs) could ameliorate lipopolysaccharide- (LPS-) induced acute lung injury (ALI) in a rat model.

**Methods:**

ALI was induced via injection of LPS. Rats were divided into three groups: (1) saline group(control), (2) LPS group, and (3) MSC + LPS group. The rats were sacrificed at 6, 24, and 48 hours after injection. Serum, bronchoalveolar lavage fluid (BALF), and lungs were collected for cytokine concentration measurements, assessment of lung injury, and histology.

**Results:**

UC-MSCs increased survival rate and suppressed LPS-induced increase of serum concentrations of pro-inflammatory mediators TNF-α, IL-1β, and IL-6 without decreasing the level of anti-inflammatory cytokine IL-10. The MSC + LPS group exhibited significant improvements in lung inflammation, injury, edema, lung wet/dry ratio, protein concentration, and neutrophil counts in the BALF, as well as improved myeloperoxidase (MPO) activity in the lung tissue. Furthermore, UC-MSCs decreased malondialdehyde (MDA) production and increased Heme Oxygenase-1 (HO-1) protein production and activity in the lung tissue.

**Conclusion:**

UC-MSCs noticeably increased the survival rate of rats suffering from LPS-induced lung injury and significantly reduced systemic and pulmonary inflammation. Promoting anti-inflammatory homeostasis and reducing oxidative stress might be the therapeutic basis of UC-MSCs.

## Background

Acute lung injury (ALI) and acute respiratory distress syndrome (ARDS) are common complications following sepsis. Lipopolysaccharide (LPS) is considered to be an important mediator of sepsis in response to gram-negative bacteria. Hence, systemic administration of LPS has been widely used as a clinically relevant model of sepsis-related ALI [1]. Despite advances in supportive care and ventilator management, mortality from ALI/ARDS remains unacceptably high. Therefore, novel effective therapies are significantly needed.

Mesenchymal stem cells (MSCs) are cells of stromal origin that can be isolated from multiple human tissues, such as bone marrow (BM), adipose tissue, skeletal muscle, synovium, gingiva, amniotic fluid, umbilical cord blood, and the umbilical cord (UC). The ability of MSCs to modulate the functions of cells associated with both innate and adaptive immune systems makes them promising therapeutic candidates in the treatment to various inflammatory diseases, including ALI/ARDS [2-4]. Recently, several studies have suggested that the administration of bone marrow- derived MSCs (BM-MSCs) in animal models of ALI can reduce systemic inflammation, ameliorate lung damage, and improve survival [5-8]. However, harvesting a patient’s BM to isolate and culture autologous MSCs cannot be done quickly enough to provide emergency treatment for acute illnesses such as ALI. Compared with the BM, the human umbilical cord-derived mesenchymal stem cells (UC-MSCs) grow more rapidly and can also secrete many types of factors to create an immunosuppressive milieu [9,10], So UC may be an ideal and practical source because of its accessibility, painless procurement from donors, lower risk of viral contamination, and lack of any ethical concerns. In this study, we investigate the therapeutic potential of UC-MSCs in a LPS-induced rat model of ALI.

## Methods

### Animal care

Male Sprague–Dawley rats (weighing 240-280 g; from Shandong University experimental animal center) were used. Animals were maintained in the animal facility at the Qilu Hospital of Shandong University. All experimental protocols were approved by the Institutional Animal Care and Use Committee at Shandong University.

### Generation and administration of UC-MSCs

UCs (n = 10, clinically normal pregnancies, approved by the Qilu hospital’s human research ethics committee) were excised and washed in a 0.1 mol/l phosphate buffer (pH 7.4) to remove excess blood. The cords were dissected and the blood vessels were removed. The remaining tissues were cut into small pieces (1–2 mm^3^) and placed in plates with low-glucose Dulbecco-modified Eagle medium (L-DMEM) (Gibco-BRL, Grand Island, NY), supplemented with 10% fetal bovine serum (FBS, Gibco-BRL), 2 ng/mL vascular endothelial growth factor (VEGF; R&D Systems, Minneapolis, MN), 2 ng/mL epidermal growth factor (EGF; R&D Systems), 2 ng/mL fibroblast growth factor (FGF; R&D Systems), 100 U/ml penicillin, and 100 μg/ml streptomycin (Gibco-BRL). Cultures were maintained at 37°C in a humidified atmosphere with 5% CO_2_. The media were changed every 3–4 d. Adherent cells proliferated from individual explanted tissues 7–12 d after initiating incubation. At this time, the small tissue pieces were removed from the culture and the adherent fibroblast-like cells were cultured to confluence, which subsequently took 2–3 weeks in culture. The cells were then trypsinized using 0.25% trypsin (Gibco-BRL) and passaged at 1 × 10^4^ cells/cm^2^ in the medium described above. The cells were used after five or more passages.

### Cell surface antigen phenotyping

Fifth- to seventh-passage cells were collected and treated with 0.25% trypsin. The cells were stained with either fluorescein isothiocyanate-conjugated or phycoerythrin-conjugated monoclonal antibodies in 100 μl phosphate buffers for 15 min at room temperature, as suggested by the manufacturer. The antibodies used were against human antigens CD29, CD34, CD44, CD45, CD73, CD90, CD105, and CD106 (SeroTec, Raleigh, NC). Cells were analyzed using flow cytometry (Cytometer 1.0, CytomicsTM FC500, Beckman Coulter). Positive cells were counted and compared to the signal of corresponding immunoglobulin isotypes.

### Differentiation capacity

To investigate the differentiation potential of the fibroblast-like cells, P4 cells were cultured under conditions appropriate for inducing the differentiation of each lineage. Cells were seeded at a density of 2x10^4^ cells/cm^2^ and the differentiation media were changed every 3–4 d. The osteogenic differentiation medium consisted of L-DMEM supplemented with 10% FBS, 0.1 μM dexamethasone, 50 mM β-glycerol phosphate, and 0.2 mM ascorbic acid (Sigma-Aldrich, St. Louis, MO). The adipogenic differentiation medium consisted of high-glucose DMEM supplemented with 0.25 mM 3-isobutyl-1-methylxanthine, 0.1 μM dexamethasone, 0.1 mM indomethacin (Sigma-Aldrich), 6.25 μg/ml insulin (PeproTech, UK), and 10% FBS (Gibco-BRL). Cells kept in the normal growth medium served as the control.

### Experimental design and LPS-induced lung injury

Briefly, rats were randomly assigned into one of three groups: saline control group, LPS group, and MSC + LPS group (n = 15 for each group). ALI was induced by the injection of LPS from *E.coli* O111:B4 (Sigma-Aldrich) (10 mg/kg intraperitoneal) and left untreated for 1 h, after which rats were given either MSCs [5 × 10^5^ cells in 300 μl of normal saline (NS)] or 300 μl of NS via injection into the tail vein. Additional experiments were done in which a human fibroblast cell line, MRC-5, was used as additional control (5 × 10^5^ cells in 300 μl of NS). Three to five rats from each group were anesthetized and sacrificed at each time point (6, 24, and 48 hours post-injection of LPS) for cytokine concentration measurements, assessment of lung injury, and histology.

Four groups of rats (n = 20 per group) were used for survival study. LPS, UCMSCs and fibroblast cells were given as described above. The rats were then allowed to recover. Mortality was recorded up to 48 hours after the treatment.

### Collection of bronchoalveolar lavage fluid (BALF) and tissue samples

Rats were euthanized and their thoraxes were opened by a midline thoracotomy. 3 ml of blood was then collected from the heart and centrifuged at 2000 rpm at 4°C for 10 min. The serum was collected and stored at −80°C for later analysis. After euthanizing the rats, the trachea was isolated and the right bronchial tube was ligated. BALF was obtained by placing a 20-gauge catheter into the trachea through which 3 ml of cold PBS was flushed back and forth three times. The BALF was centrifuged at 3000 rpm for 20 min at 4°C. The resulting cell pellet was used to determine the total cell count through the use of a counter (Beckman Coulter). A cell smear was made using Wright-Giemsa staining to confirm the neutrophil percentage. Protein concentration of the cell-free BALF from all groups was measured via Bio-Rad protein assay kit and used as an indication of endothelial and epithelial permeability. The right middle lung lobes were stored in liquid nitrogen at −80°C until subsequent analysis. The right upper lobes were used for quantifying the magnitude of pulmonary edema. The right lower lobes were used for histological evaluation.

### Lung histopathology

Paraffin-embedded lungs were cut into 5 μm thick sections and subsequently stained with hematoxylin and eosin for histological analysis. A pathologist blindly scored each lung injury using the following four categories: alveolar congestion, hemorrhage, neutrophil infiltration into the airspace or vessel wall, and thickness of alveolar wall/hyaline membrane formation. Each category was graded on a 0- to 4-point scale: 0 = no injury; 1 = injury up to 25% of the field; 2 = injury up to 50% of the field; 3 = injury up to 75% of the field; and 4 = diffuse injury [5].

### Wet-dry analysis

The right upper lobes of lungs were placed into previously-weighed microcentrifuge tubes and weighed. Lungs were then desiccated under a vacuum overnight at 80°C and weighed again. The wet lung mass was divided by the dry lung mass to give the wet-dry ratio.

### Cytokine measurement in serum

TNF-α, IL-1β, IL-6, and IL-10 serum levels of the rats was measured by enzyme-linked immunosorbent assay (ELISA) according to the manufacturer’s instructions (R&D Systems).

### Measurement of myeloperoxidase (MPO) activity

To quantify neutrophil infiltration, MPO activity in the homogenized lung tissues was measured as previously described by Jin et al. [11]. After thawing, lung tissues were homogenized in a phosphate buffer (20 mM, pH 7.4) and centrifuged at 30,000 g for 30 min. The pellet was then resuspended in a potassium phosphate buffer (50 mM, pH 6.0) with 0.5% hexadecyltrimethyl ammonium bromide. Samples were then centrifuged at 20,000 g for 15 min at 4°C. The supernatants were isolated. After addition of 0.167 mg/mL O-dianisidine hydrochloride and 0.0005% hydrogen peroxide to each sample, their absorbances were measured via spectrophotometry at 460 nm. Results were expressed as units of MPO per gram of wet tissue.

### Malondialdehyde (MDA) analysis

MDA levels in the lung tissue were used as an indicator of lipid peroxidation and were measured in tissue homogenate by the method detailed previously by Kurutas et al. [12]. In brief, 0.2 ml of the lung homogenate was mixed with 0.2 ml of 8.1% sodium dodecyl sulfate, 1.5 ml of 20% acetic acid, and 1.5 ml of 0.8% aqueous solution of thio-barbituric acid. The pH of the mixture was adjusted to 3.5 and the volume was brought up to 4.0 ml using distilled water. This mixture was then kept in a boiling water bath for 1 h. After cooling under tap water, 1.0 mL of distilled water and 5.0 ml of a mixture of n-butanol and pyridine (15:1, v/v) was added. The resulting mixture was shaken vigorously. The absorbance of the organic layer was measured via spectrophotometry at 532 nm. MDA level was expressed as nmol/mg protein.

### Heme oxygenase-1 (HO-1) activity assay

HO-1 activity was determined by the spectrophotometry of bilirubin formation, as described previously [13]. Briefly, the reaction mixture consisted of 200 μl of lung supernatant, 50 μl of liver cytosol, 20 μl of 1 mmol/L heme b solution, 200 μl of 2.75 mmol/L β-NADPH solution, and 530 μl of 2 mmol/L MgCl_2_ 100 mmol/L phosphate buffer (pH 7.4). The samples were incubated in a 37°C water bath in the dark for 1 hour. The reaction was stopped by placing the samples on ice. An NADPH-free reaction mixture was used as a baseline to which the measured concentrations were compared. The absorbance of the samples was measured by a UV/visible spectrophotometer ultrospec 2000 (Pharmacia Biotech) at 464 nm and 530 nm. The amount of bilirubin formed was calculated from the difference in absorbance measurements at 464 nm and 530 nm. The values were expressed as picomoles of bilirubin formed per milligram of protein per hour.

### Western blotting for HO-1

Lung homogenates were evaluated for HO-1 protein expression by Western blotting with primary antibodies to HO-1 (Santa Cruz Biotechnology, Santa Cruz, CA). Western blot analysis was performed as described previously [14].

### Statistical analysis

All data are presented as mean ± standard deviation. They were compared via ANOVA followed by a Student’s t-test. In the mortality study, time-to-survival data were analyzed by the Kaplan-Meier method and compared via the log-rank test. Differences between values were considered significant at *P* < 0.05.

## Results

### Characterization of the UC-MSCs and their differentiation capacity

**Figure 1:**
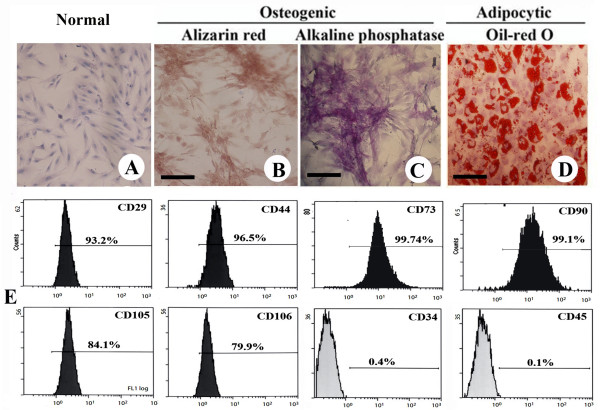
**UC-derived MSC-like cells in passaged cultures.** (**A**) H&E staining of UC-derived MSC-like cells. Osteogenic differentiation as indicated by (**B**) the formation of mineralized matrix shown by alizarin red staining and (**C**) alkaline phosphatase expression. Adipocytic differentiation was noted by the presence of broadened morphology and formation of lipid vacuoles (**D**) (positive oil-red O staining). Scale bars = 80 μm. (**E**) Immunophenotype of UC-derived MSC-like cells.

After several passages, adherent cells from UC could form a monolayer of typical fibroblastic cells (Figures 1A). Flow cytometry results showed that UC-derived cells shared most of their immunophenotype with mesenchymal stem cells, including positive expression for stromal markers (CD29, CD44, CD73, CD90, CD105, and CD106), but negative expression for hematopoietic markers (CD34 and CD45) (Figure 1E).

MSC differentiation was assessed using P4 cells. When induced to differentiate under osteogenic conditions, MSC congregation increased with increasing induction time and formed a mineralized matrix, as confirmed by alizarin red staining (Figure 1B). Most of the MSC-like cells became alkaline-phosphatase-positive by the end of 14 d (Figure 1C). No mineralized matrix was observed in the control cells kept in the normal growth medium. The spindle shape of the MSCs flattened and broadened after 1 wk of adipogenic induction. Small oil droplets gradually appeared in the cytoplasm. By the end of the second week, almost all cells contained numerous oil-red-O-positive lipid droplets (Figure 1D). The control cells maintained in the regular growth medium did not stain positive for oil red O.

### UC-MSCs treatment attenuates systemic inflammation associated with LPS

**Figure 2:**
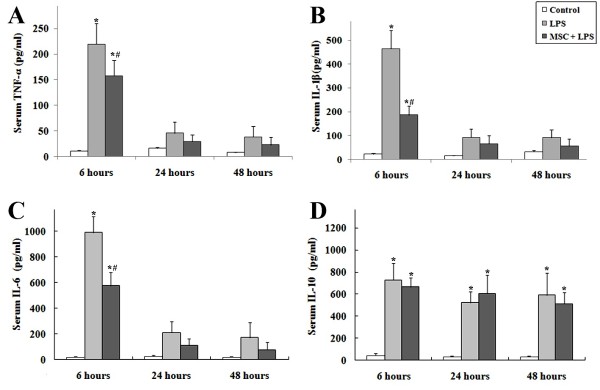
**(A-C) LPS caused a significant acute systemic inflammatory response as early as 6 hours.** Administration of UC-MSCs reduced pro-inflammatory cytokines at each time point. (**D**) LPS also caused an increase of anti-inflammatory cytokine IL-10, but this change was not altered by intravenous administration of UC-MSCs (*, p < 0.05 compared with healthy controls; #, p < 0.05 comparing LPS plus UC-MSCs with LPS alone).

Animals receiving LPS showed physical signs of systemic illness including lethargy, piloerection, and diarrhea. Serum was collected at 6, 24, and 48 hours to evaluate levels of TNF-α, IL-1β, IL-6, and IL-10 (Figure 2). LPS caused a significant acute systemic inflammatory response as evidenced by the increased serum concentrations of the pro-inflammatory mediators TNF-α, IL-1β, and IL-6. The response of pro-inflammatory cytokines reached the peak at 6 hours after injection of LPS, and decreased gradually at the 24 and 48 hour time-points. The presence of UC-MSCs reduced the increase of these three pro-inflammatory cytokines at each of the time point. LPS also caused an increase of the serum concentration of the anti-inflammatory cytokine IL-10. This change in IL-10 concentration was not altered by intravenous administration of UC-MSCs.

### UC-MSCs prevents LPS-induced ALI

#### Histology

**Figure 3:**
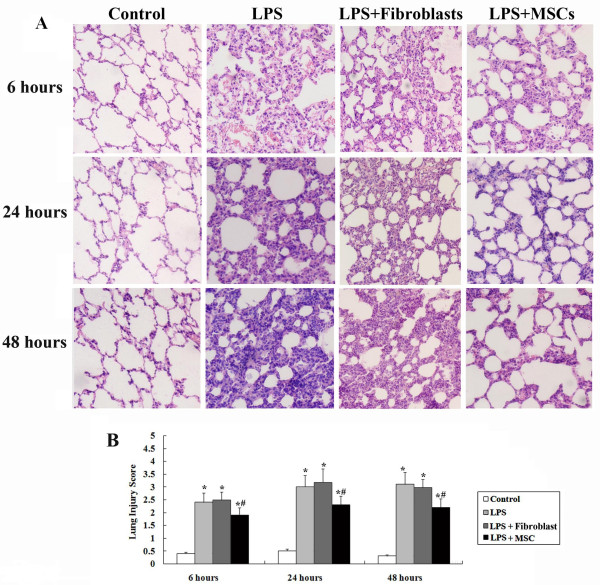
**(A) Histological analysis indicated LPS injection caused capillary expansion and congestion, as well as neutrophil infiltration into the lung tissue.** In addition, lung septae was noticeably thickened. Administration of UC-MSCs improved the lung injury at all time points. (**B**) Lung injury score decreased significantly in the MSC + LPS group at all time points (*, p < 0.05 compared with healthy controls; #, p < 0.05 comparing LPS plus UC-MSCs with LPS alone.). Fibroblast injections have no effect on improvement of both pathological morphology and lung injury score.

Six hours after intraperitoneal injection of LPS, the capillaries in the lung tissue expanded and became congested by a significant increase in neutrophils. These events peaked at the 24 hour time-point. In addition, the lung septae obviously thickened and did not show any improvement 48 hours later. MSC + LPS rats also displayed moderate injury, but the severity was significantly less compared to the LPS group at all three time points (Figure 3A). To quantify the effect of UC-MSCs on ALI, we used a 5-level score evaluation system. As shown in Figure 3B, the lung injury score was significantly lower in the MSC + LPS group at all time points. In contrast, rats given injection of human fibroblast cell line, MRC-5, had no improvement in lung injury (Figure 3A and 3B).

#### Wet-dry ratio and BALF protein concentration

**Figure 4:**
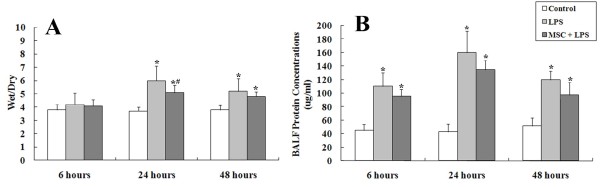
**(A) Injection of UC-MSCs reduced pulmonary edema induced by LPS.** Pulmonary edema was measured as the wet-dry ratio. (**B**): BALF protein concentration significantly increased after LPS injection and reached its peak at 24 h, while UC-MSCs caused a non-satistically significant decrease in the protein concentration. (*, p < 0.05 compared with healthy controls; #, p < 0.05 comparing LPS plus UC-MSCs with LPS alone.).

The lung wet-dry weight ratio was significantly higher at 24 h after LPS administration and slightly reduced at 48 h. However, UC-MSCs attenuated this change significantly (Figure 4A). In terms of endothelial and epithelial permeability, BALF protein concentration increased quickly after LPS injection and reached its peak at 24 hours. The MSC + LPS group exhibited relatively lower protein concentrations, but this difference was not statistically significant (Figure 4B).

#### Neutrophil infiltration in the lungs

**Figure 5:**
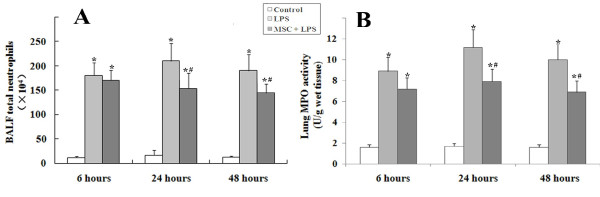
**(A) BALF neutrophil counts and (B) lung MPO activity.** Neutrophil counts and lung tissue MPO activity were significantly higher in the LPS group compared to the control group. Treatment with UC-MSCs significantly reduced the LPS-induced increase in BALF neutrophil counts and lung MPO activity at 24 and 48 hours (*, p < 0.05 compared with healthy controls; #, p < 0.05 comparing LPS plus UC-MSCs with LPS alone).

LPS caused significant increase of neutrophil counts in the BALF and MPO activity in the lung tissue at 24 and 48 h. These increases were reduced in the MSC + LPS group (Figures 5A and 5B).

#### MSC transplantation inhibited LPS-induced oxidative stress in lung parenchyma

**Figure 6:**
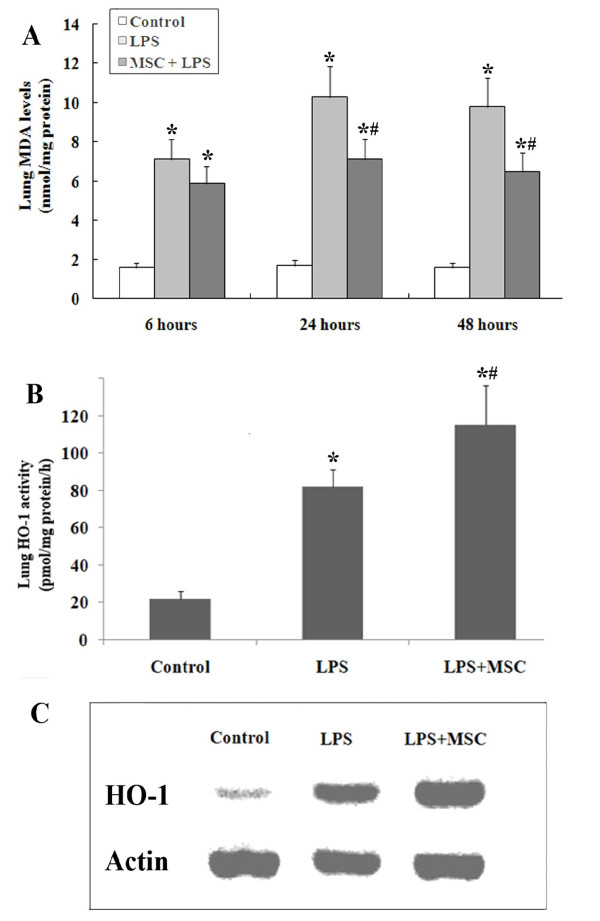
**(A) MDA levels were significantly increased in LPS-treated rats compared to the control group.** This increase was significantly reduced at 24 and 48 hours by treatment with UC-MSCs. (**B**) Activity of HO-1 in the lungs of LPS-treated rats at 24 hours. (**C**) Expression of HO-1 as measured by Western blotting analysis at 24 hours. LPS induced HO-1 protein expression and stimulated HO-1 activity in the lung tissues. Treatment with UC-MSCs further increased the expression and activity of HO-1 (*, p < 0.05 compared with healthy controls; #, p < 0.05 comparing LPS plus UC-MSCs with LPS alone).

Lung MDA levels increased markedly in the LPS group compared with the control group at each time point, whereas the increase was significantly attenuated in the MSC + LPS group as shown in Figure 6A. In the control group, the lung expression of HO-1 was very weak at 24 h. However, HO-1 expression was found to be markedly enhanced in the LPS group and even higher in the MSC + LPS group (Figure 6C). A similar observation was made in terms of lung HO-1 activity (Figure 6B).

#### MSCs improved survival rates

**Figure 7:**
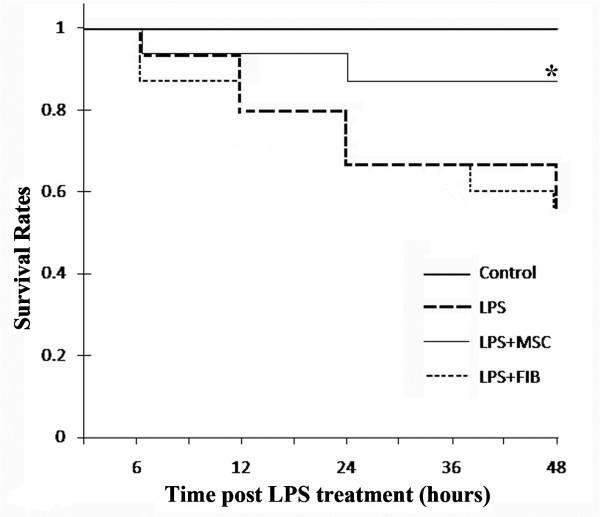
The survival rate of the LPS + MSC group over 48 hours is significantly higher than that of the LPS group (87% *vs* 60%; * p < 0.05). UC-MSCs treatment improved survival rates at all time-points.

Rats that received UC-MSCs had significantly higher rate of survival versus the LPS group (87% vs. 60%; Figure 7), in contrast, rats given fibroblast cells have no improvement in survival compared with LPS group. Treatment with UC-MSCs increased the survival rate about 20% over 48 hours. p < 0.05.

## Discussion

As shown in several animal studies over the last few years, BM-MSCs can alleviate LPS-induced acute lung injury (ALI) by restoring lung function and increasing survival rate via its anti-inflammation, anti-apoptosis, and immune regulation properties. They thus may provide a new therapy for ALI. However, since BM-MSCs decrease in number and proliferative capacity as donor age increases and are vulnerable to infection during preparation [15], it is necessary to find a new MSC source.

MSCs can be isolated from almost any tissue and organ. Its low immunogenicity and advantages in immune regulation are independent from the tissue source [16,17]. Compared to BM-MSCs, UC-MSCs have more advantages. UC-MSCs are easier to access and collect, are more secure and abundant, and exhibit higher proliferation rates [18,19]. It has been reported that UC-MSCs showed great capacity for immunomodulation, anti-inflammation, and anti-oxidation in treating lupus [20], colitis [21], bleomycin-induced pulmonary fibrosis [22] and arthritis [23]. However, little is known about UC-MSCs in the treatment of ALI.

Data from this study demonstrated that intravenous injection of UC-MSCs 1 hour after endotoxin injury clearly improved the survival rate of the rat model, significantly reduced the systemic and pulmonary inflammation, and ameliorated the pathological conditions of lung injury. The improvement of anti-inflammatory homeostasis and decrease of oxidative stress could be the key mechanisms of the treatment.

Severe endotoxemia may activate inflammatory cells and cause inflammatory reactions that lead to tissue and organ injury, dysfunction, and even death. Lung tissue is one of the most vulnerable tissues to endotoxemia. LPS can cause ALI and further develop to acute respiratory distress syndrome (ARDS) [24,25]. We created an endotoxemia rat model via the use of intraperitoneal injection of LPS, simulating sepsis-related lung injury, in order to observe the effect of UC-MSCs on acute lung injury. Experimental results showed that the rats exhibited varying degrees of lung tissue hyperemia, hemorrhage, alveolar septal thickening, infiltration of inflammatory cells, and neutrophil accumulation, which are all pathological changes associated with acute lung injury. This indicated the model was successful.

Transient inflammatory reactions are used to protect the body against infection and toxin invasion. ALI is an uncontrollable pulmonary inflammation caused by large amounts of inflammatory cells and cytokines. Under the effects of LPS, lung macrophages and neutrophils produce pro-inflammatory cytokines, like TNF-α and IL-1β, triggering the inflammatory reaction cascade [26-28]. In this study, the plasma concentrations of TNF-α, IL-1β, and IL-6 significantly increased 6 hours after intraperitoneal injection of LPS. When UC-MSCs were administrated 1 hour after LPS-induced injury, the plasma concentration of pro-inflammatory cytokines and lung inflammation decreased significantly. In vitro studies showed that BM-MSCs can reduce TNF-α and IL-6 secretion by lung macrophages via paracrine pathway or direct contact with host cells [5,6]. The difference between ALI/ARDS and normal inflammatory responses lies in the imbalance between inflammatory and anti-inflammatory activity, of which IL-10 is one of the most important anti-inflammatory cytokines. It has been reported that BM-MSCs may markedly increase the IL-10 concentration systemically and locally in LPS-induced ALI rats. In this study, the plasma IL-10 level rose markedly after intraperitoneal injection of LPS, while it didn’t change after UC-MSCs administration, which differs from other studies [5,29]. UC-MSCs administration clearly inhibits the production of pro-inflammatory cytokines TNF-α, IL-1β, and IL-6, and does not suppress the IL-10 level. Thus, it improves the homeostasis of the cytokine network and thus the balance between the inflammatory and anti-inflammatory reactions associated with ALI.

Neutrophil accumulation in the lung and pulmonary edema are two other important attributes of ALI. Neutrophils accumulated in the lung may cause mechanical obstruction of the pulmonary capillary bed, leading to microcirculation disturbance. In addition, the metabolic products of stranded and activated neutrophils can destroy the alveolar capillary barrier and increase its permeability. This causes protein-rich fluid to leak into the alveolar lumen and interstitial lung, which results in pulmonary edema [24,30]. As a proteinase highly expressed in neutrophils, MPO is the major indicator of neutrophil infiltration [31]. Compared to the LPS-injected rats, rats intravenously infused with UC-MSCs clearly reduced the amount of neutrophils in bronchoalveolar lavage fluid and MPO activity in lung tissues. Moreover, the lung wet-dry ratio results showed that the pulmonary edema improved. MSCs can produce several epithelial-specific growth factors, such as soluble paracrine factors ANG1, KGF, and HGF, which are important in ameliorating the increased lung permeability induced by LPS [32]. MSCs may also reduce the permeability of human umbilical vein endothelial cells by using VEGF to stimulate the up-regulation of vascular endothelial cell cadherin and β-catenin [33]. Although the mechanisms of pulmonary edema and neutrophil accumulation are different, the two events may not occur simultaneously [34]. Our study showed that both of these events might participate in the process by which MSCs modulate LPS-induced injury.

Oxidative stress is a sign of inflammation. Previous studies on various lung inflammation diseases confirmed that oxidative stress and oxidative damage are closely related to the development and severity of ALI/ARDS [35-37]. During ALI/ARDS, the main sources of reactive oxygen species (ROS) in lung tissue are neutrophils and macrophages. ROS-induced ALI occurs on a pathway parallel to the inflammatory reaction. MDA is the main product of lipid peroxidation and most tests define the degree of oxidative damage of the body by determining the amount of MDA [38]. This study shows that UC-MSCs significantly reduced the amount of MDA in the damaged lung tissue, indicating the redox environment in the lung improved. HO-1 is the most easily induced antioxidative enzyme in vivo, with strong antioxidative stress and cytoprotective effects [39]. UC-MSCs significantly increased the synthesis of HO-1 while reducing the amount of MDA in the lung, indicating that antioxidative stress is an important factor that is addressed when treating endotoxin-induced lung injury with UC-MSCs. Several in vitro and in vivo studies proved that MSCs can potentially regulate the redox environment. Iyer et al. found that BM-MSCs can maintain the steady-state of cysteine (Cys) and glutathione (GSH) in plasma during endotoxemia and reduce the oxidation of the Cys and GSH redox system [40]. Sun et al. confirmed that the antioxidation effect of adipose tissue derived-MSCs play an important role in ameliorating lung ischemia-reperfusion injuries [41].

To date, the mechanisms responsible for the therapeutic effects of MSCs on ALI have not been completely understood. The multi-potent property and the ability to secrete multiple paracrine factors are the potential mechanisms underlying their therapeutic use. Due to the low engraftment rates of <1% in lung injury models [8,42], recent studies consider the capacity to secrete paracrine soluble factors to be the major beneficial role. Through cell-contact-dependent and -independent mechanisms, MSCs secrete or induce multiple paracrine factors such as transforming growth factor-β, tumor necrosis factor α induced protein, IL-10, indoleamine 2, 3-dioxygenase, PGE2 to mediate immunomodulation, and keratinocyte growth factor, Angiopoietin-1 to regulate lung endothelial permeability [43]. Although we had not measured these factors except IL-10, we found reducing oxidative stress might be one of the therapeutic bases of UC-MSCs, further study is needed to understand the anti-oxidative mechanism of these cells. A recent study demonstrated that intrapulmonary delivery of human UC-MSCs attenuates acute lung injury by expanding CD4 + CD25+ Forkhead Boxp3(FOXP3) + regulatory T Cells, despite different cytokines detected, they also confirmed the balance effect of UC-MSCs on pro- and anti- inflammatory cytokines in ALI [44].

In this study, we applied human UC-MSCs to LPS-induced lung injury in a rat model. The xenogenic cell transplantation showed good therapeutic effects. Deuse et al. found that human umbilical cord lining mesenchymal stem cells had significantly lower HLA class I expression, higher production of tolerogenic TGF-β and IL-10, and showed significantly faster proliferation comparing with adult bone marrow MSCs from patients >65 years of age [45]. Because UC-MSCs have lower immunogenicity than adult BM-MSCs, human UC-MSCs can survive for a longer period of time in mice, and a single injection does not elicit a host immune response [46]. Additionally, a one-year long continuous study of treating Parkinson’s disease in rats with human UC-MSCs confirmed its safety and efficacy [47]. Chen et al. found that UC-MSCs had higher endothelial differentiation potential than BM-MSCs. Therefore, UC-MSCs are more favorable choice than BM-MSCs for neovascularization of engineered tissues [48]. These studies provide a foundation for potentially treating human diseases using UC-MSCs in future.

## Conclusions

In summary, this study demonstrated that intravenous injection of UC-MSCs clearly increased the survival rate of rats suffering from LPS-induced lung injuries and significantly reduced systemic and pulmonary inflammation. Promoting anti-inflammatory homeostasis and reducing oxidative stress may be the therapeutic basis of UC-MSCs for this disease model.

## Abbreviations

MSCs: Mesenchymal stem cells; UC-MSC: Umbilical cord-derived mesenchymal stem cells; ALI: Acute lung injury; ARDS: Acute respiratory distress syndrome; MPO: Myeloperoxidase; MDA: Malondialdehyde; HO-1: Heme Oxygenase-1; BM: Bone marrow; UC: Umbilical cord; BALF: Bronchoalveolar lavage fluid; ROS: Reactive oxygen species; Cys: Cysteine; GSH: Glutathione.

## Competing interests

The authors state that they have no conflict of interest to disclose.

## Authors’ contributions

All authors read and approved the final manuscript. JL and DL participated in the experimental work and drafting the manuscript. FW and JL designed the study and drafted the manuscript, XL and ST participated in animal experiments.

## References

[B1] Wang HM Bodenstein M Markstaller K Overview of the pathology of three widely used animal models of acute lung injury Eur Surg Res 2008 40 305 316 10.1159/000121471 18349543

[B2] Weil BR Markel TA Herrmann JL Abarbanell AM Kelly ML Meldrum DR Stem cells in sepsis Ann Surg 2009 250 19 27 10.1097/SLA.0b013e3181a77b9c 19561461

[B3] Nauta AJ Fibbe WE Immunomodulatory properties of mesenchymal stromal cells Blood 2007 110 3499 3506 10.1182/blood-2007-02-069716 17664353

[B4] Weiss DJ Sueblinvong V Cell therapy approaches for lung diseases:current status Curr Opin Phamacol 2009 9 268 273 10.1016/j.coph.2009.03.002 PMC4201364 19349209

[B5] Gupta N Su X Popov B Lee JW Serikov V Matthay MA Intrapulmonary delivery of bone marrow-derived mesenchymal stemcells improves survival and attenuates endotoxin-induced acute lung injury in mice J Immunol 2007 179 1855 1863 17641052 10.4049/jimmunol.179.3.1855

[B6] Xu J Woods CR Mora AL Joodi R Brigham KL Iyer S Rojas M Prevention of endotoxin-induced systemic response by bonemarrow-derived mesenchymal stem cells in mice Am J Physiol Lung Cell Mol Physiol 2007 293 L131 L141 10.1152/ajplung.00431.2006 17416739

[B7] Xu J Qu J Cao L Sai Y Chen C He L Yu L Mesenchymal stem cell-based angiopoietin-1 gene therapy for acute lung injury induced by lipopolysaccharide in mice J Pathol 2008 214 472 481 18213733 10.1002/path.2302

[B8] Rojas M Xu J Woods CR Mora AL Spears W Roman J Brigham KL Bone marrow-derived mesenchymal stem cells in repair of the injured lung Am J Respir Cell Mol Biol 2005 33 145 152 10.1165/rcmb.2004-0330OC 15891110 PMC2715309

[B9] Lu LL Liu YJ Yang SG Zhao QJ Wang X Gong W Han ZB Xu ZS Lu YX Liu D Chen ZZ Han ZC Isolation and characterization of human umbilical cord mesenchymal stem cells with hematopoiesis-supportive function and other potentials Haematologica 2006 91 1017 1026 16870554

[B10] Fong CY Gauthaman K Cheyyatraivendran S Lin HD Biswas A Bongso A Human umbilical cord Wharton’s jelly stem cells and its conditioned medium support hematopoietic stem cell expansion ex vivo J Cell Biochem 2012 113 658 668 10.1002/jcb.23395 21976004

[B11] Jin SW Zhang L Lian QQ Liu D Wu P Yao SL Ye DY Posttreatment with aspirin-Triggered lipoxin A4 analog attenuates lipopolysaccharide-induced acute lung injury in mice: The role of heme oxygenase-1 Critical Care and Trauma 2007 104 369 377 10.1213/01.ane.0000252414.00363.c4 17242094

[B12] Kurutas EB Cetinkaya A Bulbuloglu E Kantarceken B Effects of antioxidant therapy on leukocyte myeloperoxidase and Cu/Zn-superoxide dismutase and plasma malondialdehyde levels in experimental colitis Mediators Inflamm 2005 6 390 394 16489261 10.1155/MI.2005.390 PMC1533903

[B13] Pang QF Zhou QM Zeng S Dou LD Ji Y Zeng YM Protective effect of heme oxygenase-1 on lung injury induced by erythrocyte instillation in rats Chin Med J 2008 121 1688 1692 19024100

[B14] Ge ZJ Jiang GJ Zhao YP Wang GX Yong FT Systemic perfluorohexane attenuates lung injury induced by lipopolysaccharide in rats: the role of heme oxygenase-1 Pharmacological Report 2010 62 170 177 10.1016/s1734-1140(10)70254-1 20360627

[B15] Stolzing A Jones E McGonagle D Scutt A Age-related changes in human bone marrow-derived mesenchymal stem cells: consequences for cell therapies Mech Ageing Dev 2008 129 163 173 10.1016/j.mad.2007.12.002 18241911

[B16] Suzdaltseva YG Burunova VV Vakhrushev IV Cheglakov IB Yarygin KN In vitro comparison of immunological properties of cultured human mesenchymal cells from various sources Bull Exp Biol Med 2008 145 228 231 10.1007/s10517-008-0057-y 19023976

[B17] Weiss ML Anderson C Medicetty S Seshareddy KB Weiss RJ VanderWerff I Troyer D McIntosh KR Immune properties of human umbilical cord wharton’s jelly-derived cells Stem Cells 2008 26 2865 2874 10.1634/stemcells.2007-1028 18703664

[B18] Secco M Zucconi E Vieira NM Fogaca LL Cerqueira A Carvalho MD Jazedje T Okamoto OK Muotri AR Zatz M Multipotent stem cells from umbilical cord: Cord is richer than blood! Stem Cells 2008 26 146 150 10.1634/stemcells.2007-0381 17932423

[B19] Baksh D Yao R Tuan RS Comparison of proliferative and multilineage differentiation potential of human mesenchymal stem cells derived from umbilical cord and bone marrow Stem Cells 2007 25 1384 1392 10.1634/stemcells.2006-0709 17332507

[B20] Sun L Wang D Liang J Zhang H Feng X Wang H Hua B Liu B Ye S Hu X Xu W Zeng X Hou Y Gilkeson GS Silver RM Lu L Shi S Umbilical cord mesenchymal stem cell transplantation in severe and refractory systemic lupus erythematosus Arthritis Rheum 2010 62 2467 2475 10.1002/art.27548 20506343

[B21] Liang L Dong C Chen X Fang Z Xu J Liu M Zhang X Gu DS Wang D Du W Zhu D Han ZC Human umbilical cord mesenchymal stem cells ameliorate mice trinitrobenzene sulfonic acid (TNBS)-induced colitis Cell Transplant 2011 20 1395 1408 10.3727/096368910X557245 21396175

[B22] Moodley Y Atienza D Manuelpillai U Samuel CS Tchongue J IIancheran S Boyd R Trounson A Human umbilical cord mesenchymal stem cells reduce fibrosis of bleomicin-induced lung injury Am J Pathol 2009 175 303 313 10.2353/ajpath.2009.080629 19497992 PMC2708816

[B23] Liu YY MUR Wang S Long L Liu X Sun J Guo JJ Zhang XP Guo J Yu P Li CL Liu XY Huang ZY Wang DP Li H GU ZF Liu B Li ZG Therapeutic potential of human umbilical cord mesenchymal stem cells in the treatment of rheumatoid arthritis Arthritis Res Ther 2010 12 R210 10.1186/ar3187 21080925 PMC3046518

[B24] Abraham E Neutrophils and acute lung injury Crit Care Med 2003 31 S195 S199 10.1097/01.CCM.0000057843.47705.E8 12682440

[B25] Tsushima K King LS Aggarwal NR Gorordo AD D’Alessio FR Kubo K Acute lung injury review Inter Med 2009 48 621 630 10.2169/internalmedicine.48.1741 19420806

[B26] Rojas M Woods CR Mora AL Xu J Brigham KL Endotoxin-induced lung injury in mice: structural, functional, and biochemical responses Am J Physiol Lung Cell Mol Physiol 2005 288 L333 L341 15475380 10.1152/ajplung.00334.2004

[B27] Iyer SS Co C Rojas M mesenchymal stem cells and inflammatory lung diseases Panminerva Med 2009 51 5 16 19352305

[B28] Bhatia M Moochhala S Role of inflammation mediators in the pathophysiology of acute respiratory distress syndrome J Pathol 2004 202 145 156 10.1002/path.1491 14743496

[B29] Nemeth K Leelahavanichkul A Yuen PS Yuen PST Mayer B Parmelee A Doi K Robey PG Leelahavanichkul K Koller BH Brown JB Hu X Jelinek I Star RA Mezey E Bone marrow stromal cells attenuate sepsis via prostaglandin E2-dependent reprogramming of host macrophages to increase their interleukin-10production Nat Med 2009 15 42 49 10.1038/nm.1905 19098906 PMC2706487

[B30] Grommes J Soehnlein O Contribution of neutrophils to acute lung injury Mol Med 2011 17 293 307 21046059 10.2119/molmed.2010.00138 PMC3060975

[B31] Haegens A Heeringa P van Suylen RJ Aratani Y O’Donoghue RJ Mutsaers SE Mossman BT Wouters EF Vernooy JH Myeloperoxidase deficiency attenuates lipopolysaccharide -induced acute lung inflammation and subsequent cytokine and chemokine production J Immunol 2009 182 7990 7996 10.4049/jimmunol.0800377 19494324

[B32] Yagi H Soto-Gutierrez A Kitagawa Y Tilles AW Tompkins RG Yarmush ML Bone mesenchymal stromal cells attenuate ogan injury induced by LPS and burn Cell Transpant 2010 19 823 830 10.3727/096368910X508942 PMC2957544 20573305

[B33] Pati S Khakoo AY Zhao J Jimenez F Gerber MH Harting M Redell JB Grill R Matsuo Y Guha S Cox CS Reits MS Holcomb JB Dash PK Human mesenchymal stem cells inhibit vascular permeability by modulating vascular endothelial cadherin/β-catenin signaling Stem Cells Dev 2010 20 1 13 20446815 10.1089/scd.2010.0013 PMC3128758

[B34] Chignard M Balloy V Neutrophil recruitment and increased permeability during acute lung injury induced by lipopolysaccharide Am J Physiol Lung Cell Mol Physiol 2000 279 L1083 L1090 11076798 10.1152/ajplung.2000.279.6.L1083

[B35] Iyer SS Jones DP Brigham M Oxidation of plasma cysteine/cystine redox state in endotoxin-induced lung injury Am J Respir Cell Mol Biol 2009 40 90 98 18664641 10.1165/rcmb.2007-0447OC PMC2606950

[B36] Laurent T Markert M Feihl F Schaller MD Perret C Oxidant-antioxidant balance in granulocytes during ARDS.Effect of N-acetylcysteine Chest 1996 109 163 166 10.1378/chest.109.1.163 8549180

[B37] Metnitz PG Barents C Fischer M Fridrich P Steltzer H Druml W Antioxidant status in patient with acute respiratory syndrome Intensive Care Med 1999 25 180 185 10.1007/s001340050813 10193545

[B38] Tahara M Nakayama M Jin MB Fujita M Suzuki T Taniguchi M Shimamura T Furukawa H Todo S A radical scavenger, edaravone, protects canine kidneys from ischemia-reperfusion Injury after 72 hours of cold preservation and autotransplantation Transplantation 2005 80 213 221 10.1097/01.TP.0000165092.07375.C9 16041266

[B39] Fredenburgh LE Perrella MA Mitsialis SA The role of heme oxygenase-1 in pulmonary disease Am J Respir Cell Mol Biol 2007 36 158 165 16980551 10.1165/rcmb.2006-0331TR PMC2176110

[B40] Iyer SS Torres-Gonzalez E Neujahr DC Kwon M Brigham KL Jones DP Mora AL Rojas M Effect of Bone Marrow-Derived Mesenchymal Stem Cells on Endotoxin-Induced Oxidation of Plasma Cysteine and Glutathione in Mice Stem Cells International 2010 2010 868076 21048855 10.4061/2010/868076 PMC2963315

[B41] Sun CK Yen CH Lin YC Tsai TH Chang LT Kao YH Chua S Fu M Ko SF Leu S Yip HK Autologous Transplantation of Adipose-Derived Mesenchymal Stem Cells Markedly Reduced Acute Ischemia-Reperfusion Lung Injury in a Rodent Model J Transl Med 2011 9 118 10.1186/1479-5876-9-118 21781312 PMC3155151

[B42] Kotton DN Fabian AJ Mulligan RC Failure of bone marrow to reconstitute lung epithelium Am J Respir Cell Mol Biol 2005 33 328 334 10.1165/rcmb.2005-0175RC 15961722 PMC2715341

[B43] Lee JW Fang X Krasnodembskaya A Howard JP Matthay MA Mesenchymal stem cells for acute lung injury: role of paracrine soluble factors Stem Cells 2011 29 913 919 10.1002/stem.643 21506195 PMC3293251

[B44] Sun J Han ZB Liao WB Yang SG Yang ZX Yu JX Meng L Wu R Han ZC Intrapulmonary delivery of human umbilical cord mesenchymal stem cells attenuates acute lung injury by expanding CD4 + CD25+ Forkhead Boxp3 (FOXP3) + regulatory T cells and balancing anti and pro-inflammatory factors Cell Physiol Biochem 2011 27 587 596 10.1159/000329980 21691076

[B45] Deuse T Stubbendorff M Tang-Quan K Phillips N Kay MA Eiermann T Phan TT Volk HD Reichenspurner H Robbins RC Schrepfer S Immunogenicity and immunomodulatory properties of umbilical cord lining mesenchymal stem cells Cell Transplant 2011 20 655 667 10.3727/096368910X536473 21054940

[B46] Nakamizo A Marini F Amano T Khan A Studeny M Gumin J Chen J Hentschel S Vecil G Dembinski J Andreeff M Lang FF Human bone marrow-derived mesenchymal stem cells in the treatment of gliomas Cancer Res 2005 65 3 307 10.1158/0008-5472.CAN-04-1874 15833864

[B47] Xiong N Cao X Zhang Z Huang J Chen C Zhang Z Jia M Xiong J Liang Z Sun S Lin Z Wang T Long-term efficacy and safety of human umbilical cord mesenchymal stromal cells in rotenone-induced hemiparkinsonian rats Biol Blood Marrow Transplant 2010 16 1519 1529 10.1016/j.bbmt.2010.06.004 20542126

[B48] Chen MY Lie PC Li ZL Wei X Endothelial differentiation of Wharton’s jelly-derived mesenchymal stem cells in comparison with bone marrow-derived mesenchymal stem cells Exp Hematol 2009 37 629 640 10.1016/j.exphem.2009.02.003 19375653

